# The maturation of exploratory behavior in adolescent *Mus spicilegus* on two photoperiods

**DOI:** 10.3389/fnbeh.2022.988033

**Published:** 2022-11-04

**Authors:** Noah G. Cryns, Wan Chen Lin, Niloofar Motahari, Oliver J. Krentzman, Weihang Chen, George Prounis, Linda Wilbrecht

**Affiliations:** ^1^Department of Molecular and Cellular Biology, University of California, Berkeley, Berkeley, CA, United States; ^2^Helen Wills Neuroscience Institute, University of California, Berkeley, Berkeley, CA, United States; ^3^Department of Psychology, University of California, Berkeley, Berkeley, CA, United States

**Keywords:** dispersal, seasonality, adolescence, open field, novel object, wild mouse

## Abstract

Dispersal from the natal site or familial group is a core milestone of adolescent development in many species. A wild species of mouse, *Mus spicilegus*, presents an exciting model in which to study adolescent development and dispersal because it shows different life history trajectory depending on season of birth. *M. spicilegus* born in spring and summer on long days (LD) disperse in the first 3 months of life, while *M. spicilegus* born on shorter autumnal days (SD) delay dispersal through the wintertime. We were interested in using these mice in a laboratory context to compare age-matched mice with differential motivation to disperse. To first test if we could find a proxy for dispersal related behavior in the laboratory environment, we measured open field and novel object investigation across development in *M. spicilegus* raised on a LD 12 h:12 h light:dark cycle. We found that between the first and second month of life, distance traveled and time in center of the open field increased significantly with age in *M. spicilegus*. Robust novel object investigation was observed in all age groups and decreased between the 2nd and 3rd month of life in LD males. Compared to male C57BL/6 mice, male *M. spicilegus* traveled significantly longer distances in the open field but spent less time in the center of the field. However, when a novel object was placed in the center of the open field, Male *M. spicilegus*, were significantly more willing to contact and mount it. To test if autumnal photoperiod affects exploratory behavior in *M. spicilegus* in a laboratory environment, we reared a cohort of *M. spicilegus* on a SD 10 h:14 h photoperiod and tested their exploratory behavior at P60-70. At this timepoint, we found SD rearing had no effect on open field metrics, but led to reduced novel object investigation. We also observed that in P60-70 males, SD reared *M. spicilegus* weighed less than LD reared *M. spicilegus*. These observations establish that SD photoperiod can delay weight gain and blunt some, but not all forms of exploratory behavior in adolescent *M. spicilegus*.

## Introduction

Exploratory behavior during adolescence is a topic of great interest to psychology, psychiatry, and public health as well as integrated biology. While exploratory behavior may serve many functions, we were particularly interested in identifying exploratory behavior in rodents in a laboratory environment that could be related to natal dispersal.

Natal dispersal is a dramatic behavior observed in most vertebrates that often occurs during adolescence. Dispersal occurs when animals, typically males, but sometimes females, leave their site of birth (or hatching) to roam more broadly or to inhabit a new site where reproduction will occur. By definition, dispersing individuals increase their spatial exploration and with this exploration they encounter new and potentially threatening novel objects in their environment. Natal dispersal typically occurs before reproductive maturity, but not in all species. Hypotheses for the ultimate evolutionary causes of dispersal include competition for mates, competition for resources, and inbreeding avoidance (Greenwood and Harvey, 1982; Pusey and Wolf, 1996; Alonso et al., 1998; Long et al., 2008).

It is not well understood what proximate neural mechanisms drive natal dispersal on an acute timescale, although attainment of sufficient weight, gonadal and adrenal hormones are thought to play a role (Howard, 1960; Holekamp, 1984; O’Riain et al., 1996; Dufty and Belthoff, 2001; Duckworth, 2009; Duckworth and Sockman, 2012). External triggers may include social factors such as parental and sibling aggression (Gerlach, 1990). Dispersal in some species is regulated by photoperiodism, where day length can modulate physiological factors to trigger dispersal (Walton et al., 2011).

*Mus spicilegus* is a wild mouse species of particular interest to studies of adolescent development due to its seasonally dependent life history as well as its interesting mound building behavior (Poteaux et al., 2008; Simeonovska-Nikolova and Mehmed, 2009; Tong and Hoekstra, 2012; Couger et al., 2018). *M. spicilegus* are primarily found in agricultural ecosystems throughout Eastern Europe. Depending on the time of year in which they are born, *M. spicilegus* have different timelines for dispersal and mating as well as different social structures. Mice born in spring and early summer, a long day (LD) cohort, disperse from their natal nest and mate after 2–3 months; they do not live through the winter and have shorter lives compared to their autumnal-born counterparts (Gouat et al., 2003b; Poteaux et al., 2008). Conversely, the autumnal or short day (SD) cohort, born in late summer and fall, cooperatively build large, earthen mounds made from foraged materials in which they overwinter with related kin; delaying dispersal and reproduction 6–8 months until the following spring (Poteaux et al., 2008; Szenczi et al., 2011; Csanády et al., 2020). Thus, mice born in these different seasons have considerably different life trajectories. Based on these observations in the wild, we postulated that dispersal in *M. spicilegus* could be regulated by photoperiod and sought to test effects of photoperiod on exploration in a laboratory environment. We postulated that *M. spicilgeus* reared on LD light would express motivation to disperse during the first 2–4 months of life while those reared on SD light would not express the same level of motivation in this time frame. In this initial study we decided to use measures of exploratory behavior that are commonly assessed in domesticated rodents in the laboratory environment but that also may be putatively related to dispersal. We reasoned that if these behaviors served as a reliable proxy metric for the motivation to disperse, they would show A) increases with development in the first 2–4 months of life in LD light cycles, and B) relative suppression in SD reared compared to LD reared *M*. *spicilegus*.

To test (A) we first measured distance traveled in an open field and novel object investigation behavior in male and female *M. spicilegus* born on a LD 12 h day length. For reference, we also compared male *M. spicilegus* exploratory behavior to male C57 BL/6 mice. To test (B), we examined if a SD rearing reduced the expression of exploratory behavior at a peak adolescent timepoint in male *M. spicilegus*.

## Materials and methods

### Animals

Male and female *M. spicilegus* mice (derived from Kalomoyevka, Ukraine) were maintained as a colony from a limited founder population obtained from France (courtesy of the lab of François Bonhomme).

*M. spicilegus* were reared in one of two different photoperiods meant to mimic spring/summer and autumnal conditions. One cohort of *M. spicilegus* was maintained on a 12 h:12 h light:dark cycle (LD) mimicking spring/summer conditions (12 h day length is commonly employed in animal facilities and reflects daylight hours first reached in March in Ukraine. A limitation of this study is that 12 h daylight is also experienced in Ukraine in September). A second cohort of *M. spicilegus* was reared on a shorter 10:14 h light:dark cycle (SD) mimicking autumnal conditions (day length is 10 h in late October in Ukraine). To attain mice born on the 10:14 h light cycle, some dams were moved to this light cycle pregnant and others bred on this light cycle. To study weight at P60-79 on the LD and SD light cycles, we used data from mice who ran through the battery of tasks as well as data from mice who were only weighed.

All mice were weaned at P21 and housed with 2–3 same-sex siblings and nesting material. For age comparison, LD reared mice were tested in 4 age group bins: P21-33; P35-45; P60-79; P80-125. Our age bins were informed by pubertal ages in C57 BL/6 mice who are reaching the first estrus ∼P35 and breeding ∼P60 and field work in *M. spicilegus* that suggests dispersal occurs 2–3 months after birth on LD photoperiods (Lafaille et al., 2015). Our largest sample came from LD reared male *M. spicilegus*: P21-P33 (*n* = 18), P35-P45 (*n* = 11), P60-P79 (*n* = 29), and P80-P125 (*n* = 11). Female LD reared *M. spicilegus* were tested across a comparable age range and binned into the same four age groups: P21-P33 (*n* = 7), P35-P45 (*n* = 7), P60-P79 (*n* = 10), and P80-P125 (*n* = 12). Male C57 BL/6 were tested across the same age range: P21-P33 (*n* = 9), P35-P45 (*n* = 15), P60-P79 (*n* = 9), and P80-P125 (*n* = 8). Male C57BL/6 mice were housed on a 12 h:12 h reverse light cycle. We did not study female C57 BL/6 mice.

All behavioral procedures were approved by the Animal Care and Use Committee at The University of California, Berkeley.

### Open field

**FIGURE 1:**
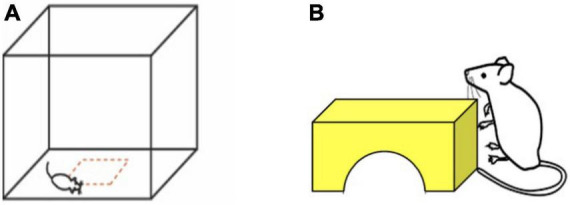
Open field and novel object investigation were used to measure exploratory behavior in the laboratory environment. **(A)** Open field area with center area defined in red. We measured distance traveled in the arena and time in center in a 15 min test. **(B)** Novel object investigation. We measured time contacting object and time on top of the object in a 5 min test. The shape, color, and size of the object was kept consistent.

Mice were placed in the center of a transparent acrylic arena with 42 cm by 42 cm floor dimensions, 30.5 cm high walls, and a removable, perforated lid (Figure 1A). The arena was housed within a sound-attenuated chamber (Med Associates; Fairfax, VT) with lights and a fan in the interior. Mice were allowed to explore the arena undisturbed for 15 min. Movement was monitored in the arena using infrared beam brakes (Versamax, AccuScan Instruments; Columbus, OH). Behavioral metrics analyzed included total distance traveled (cm) and time in center (center defined as > 7.875 cm from the walls of the arena). The arena was wiped down with 70% ethanol and allowed to dry before adding each animal. Mice were only tested once.

### Novel object investigation

Immediately following testing in the open field, mice were momentarily covered with a large plastic cup and gently moved to one corner of the arena. A 2 inch high yellow block bridge was placed in the center of the arena facing the corner in which the mouse was covered (Figure 1B). The cup was then removed, the doors to the sound-attenuated chamber were closed, and mice were given 5 min to explore the open field arena with the novel object present. Mice were removed from the open field arena after 5 min, weighed, and returned to their home cage. The placement of the object in the center of the arena, as well as novelty of the object both likely contribute to the challenge of this task. Novel object investigation behavior was analyzed using the Boris animal tracking software (Friard and Gamba, 2016). We scored time contacting the object and time on top of the object.

### Statistical analyses

Statistical comparisons were made using Graphpad Prism software. We examined main effects of age and sex (or age and species) and their interactions using 2 way ANOVA. We used Sidak’s multiple comparison test to compare age groups within sex or species and report adjusted *p*-values. LD and SD males were compared using Mann-Whitney U-test.

## Results

### Long day male and female *Mus spicilegus* show increases in exploratory behavior in the open field during the first 3 months of life

**FIGURE 2:**
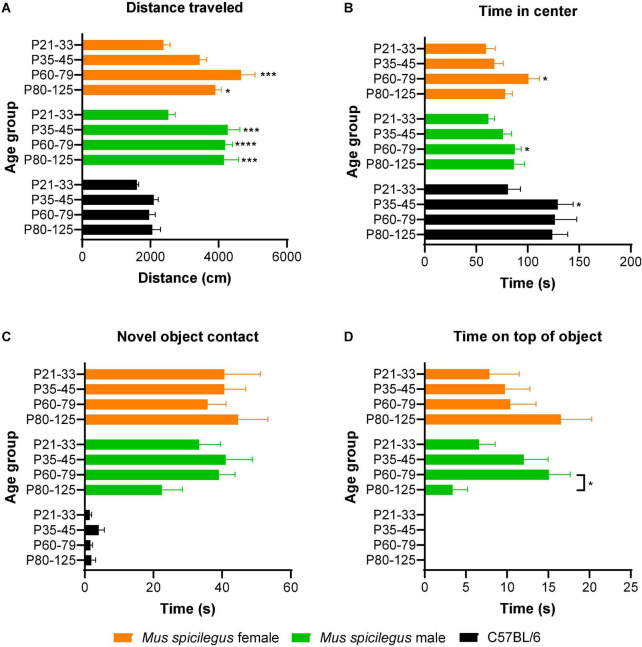
Male and female *Mus spicilegus* reared on LD photoperiod show comparable adolescent development of exploratory behavior that differs from that seen in C57BL/6 males. **(A)** Distance traveled in the open field. Male *M. spicilegus* showed a significant increase in distance traveled in the open field in the transition between the end of the first month (P21-33) and second month of life (P35 +) (*P* < 0.001). In female *M. spicilegus*, there was a significant increase in distance traveled between P21-P33 and P60-P79 (*p* < 0.0001). No main effect of sex was observed. Comparisons between males of two species showed a significant main effect of age as well as species on distance traveled (age: *p* = 0.0002; species: *p* < 0.0001). Male *M. spicilegus* traveled more than C57 BL/6 males in the open field (species: *p* < 0.0001). No significant changes with age were observed in this metric in C57 BL/6 males. **(B)** Time in center of open field. There was no effect of sex but a significant effect of age on time in center of the open field in *M. spicilegus*. Male and female *M. spicilegus* showed a significant increase in time in center of the open field in the transition between the end of the first month (P21-33) and end of the second month of life (P60-79) (*P* < 0.05). No main effect of sex was observed. Comparisons between males of two species showed a significant main effect of age as well as species on time in the center (age: *p* = 0.007; species: *p* < 0.0001). C57 Bl/6 showed a gain in this metric from P21-33 to P35-45 *P* = 0.03). **(C)** Novel object contact. There was no significant main effect of sex or age on novel object contact in *M. spicilegus*. Species comparison showed a significant main effect of species but not age on novel object contact (age: *p* = 0.37; species: *p* < 0.0001). **(D)** Time on top of novel object (mounting). There was no significant main effect of sex but a significant effect of age on time on top of novel object in *M. spicilegus* groups. Male *M. spicilegus* showed a significant decrease in time spent on top of the novel object between P60-P79 and P80-P125 (*p* = 0.04). While *M. spicilegus* readily mounted the object even at the youngest ages, male C57/Bl/6 largely did not express this behavior in the ages sampled (age: *p* = 0.11; species: *p* < 0.0001). *p < 0.05, ***p < 0.001, ****p < 0.0001 (All * without lines are relative to P21-33 group).

In our first experiment, LD male and female *M. spicilegus* underwent open-field testing followed by a novel object investigation test. Each individual was tested only once. We found a significant main effect of age but not of sex on distance traveled [two-way ANOVA, age: *F*(3, 97) = 14.9, *p* < 0.0001; sex: *F*(1, 97) = 0.70, *p* = 0.41] (Figure 2A). We found no significant interaction between age and sex [*F*(3, 97) = 1.44, *p* = 0.24]. *Post hoc* comparisons revealed that distance traveled was significantly lower at P21-P33 compared to older ages in males and females (21-33 vs. P35-P45: male *p* = 0.0002, female *p* = 0.31; P21-33 vs. P60-79: male *p* < 0.0001, female *p* < 0.0001; P21-33 vs. P80-125: male *p* = 0.0006, female *p* = 0.018). Other age comparisons were non-significant. In the heavily sampled P60-79 male group, there was no significant linear relationship between weight and distance traveled (*R*^2^ = 0.01, *p* = 0.78).

There was a significant main effect of age but not of sex on time spent in the center of the open field [two-way ANOVA, age: *F*(3, 97) = 5.57, *p* = 0.001; sex: *F*(1, 97) = 0.06, *p* = 0.81], and no significant interaction between age and sex [*F*(3, 97) = 0.8, *p* = 0.50] (Figure 2B). *Post hoc* comparisons revealed there was a significant increase in time in center between P21-33 and P60-79 (male *p* = 0.03, female *p* = 0.04) (Figure 2B). There were no significant differences in time spent in the center of the open field between other groups (*p* > 0.14). In the heavily sampled P60-79 male group, there was no significant linear relationship between weight and time in center (*R*^2^ = 0.02, *p* = 0.34).

There was no significant main effect of age or sex on time spent contacting the novel object [two-way ANOVA, age: *F*(3, 97) = 0.29, *p* = 0.83; sex: *F*(1, 97) = 1.49, *p* = 0.23] (Figure 2C). There was no interaction between age and sex [two-way ANOVA: *F*(3, 97) = 1.28, *p* = 0.29] (Figure 2C). In the heavily sampled P60-79 male group there was no significant linear relationship between weight and novel object contact (*R*^2^ = 0.04, *p* = 0.66).

There was no significant main effect of age or sex on time on top of the novel object [two-way ANOVA, age: *F*(3, 97) = 1.05, *p* = 0.37; sex: *F*(1, 97) = 0.63, *p* = 0.43] (Figure 2D). There was a significant interaction between age and sex [two-way ANOVA: *F*(3, 97) = 3.16, *p* = 0.028] (Figure 2D). *Post hoc* comparisons revealed in males that the P60-P79 group spent significantly greater time on top of the novel object than the P80-P125 group (*p* = 0.04). *Post hoc* comparisons showed no significant difference between other age groups in metrics of time on top of the novel object (*p* > 0.12). In the heavily sampled P60-79 male group, there was no significant linear relationship between weight and distance traveled (*R*^2^ < 0.01, *p* = 0.99).

### Male *Mus spicilegus* exploratory behavior differs from C57BL/6

Next we compared the open field and novel object investigation performance of C57BL/6 males with *M. spicilegus* males across development. We ran a two-way ANOVA to assess the effect of age and species on the four exploratory metrics (distance traveled, time in center, time contacting object and time on top of the object).

*M. spicilegus* males traveled further in the open field than C57BL/6 males (Figure 2A). There was a significant main effect of species as well as age on distance traveled [two-way ANOVA, age: *F*(3, 102) = 7.35, *p* = 0.0002; species: *F*(1, 102) = 87.43, *p* < 0.0001] (Figure 2A). There was no significant interaction between age and species [two-way ANOVA: *F*(3, 102) = 2.56, *p* = 0.06] (Figure 2A).

Conversely, male C57BL/6 spent more time in the center of the open field than male *M. spicilegus* at all age groups (Figure 2B). There was a significant main effect of species as well as age on time in the center [two-way ANOVA, age: *F*(3, 102) = 4.26, *p* = 0.007; species: *F*(1, 102) = 20.0, *p* < 0.0001] (Figure 2B). There was no significant interaction between age and species [two-way ANOVA: *F*(3, 102) = 0.750, *p* = 0.53] (Figure 2B). *Post hoc* comparisons revealed that within the C57 BL/6 males, there was a significant increase in time in center between P21-33 and P35-45 (*p* = 0.03).

*M. spicilegus* males contacted the novel object more than male C57BL/6 for all 4 age bins (Figure 2C). There was a significant main effect of species but not age on novel object contact [two-way ANOVA, age: *F*(3, 102) = 1.06, *p* = 0.37; species: *F*(1, 102) = 58.4, *p* < 0.0001] (Figure 2C). There was no significant interaction between age and species [two-way ANOVA: *F*(3, 102) = 0.80, *p* = 0.50] (Figure 2C).

No C57BL/6 ever climbed on top of the novel object whereas a majority of the M. spicilegus did (Figure 2D). There was a significant main effect of species but not age on time spent on top of the novel object [two-way ANOVA, age: *F*(3, 102) = 2.06, *p* = 0.11; species: *F*(1, 102) = 26.1, *p* < 0.0001] (Figure 2D). There was no significant interaction between age and species [two-way ANOVA: *F*(3, 102) = 2.06, *p* = 0.11] (Figure 2D).

### In P60-79 males, short day photoperiod rearing affected weight and novel object investigation

**FIGURE 3:**
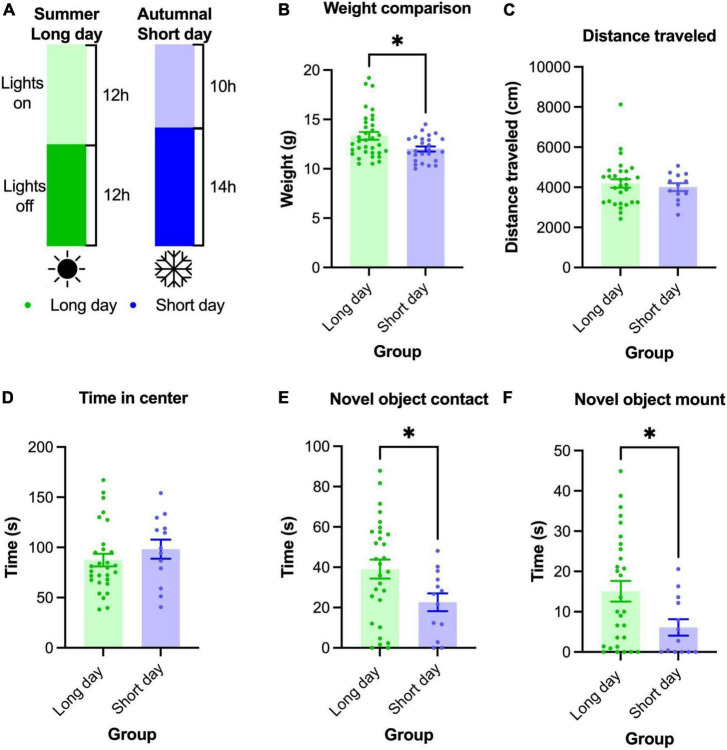
Adolescent (P60-P70) male *M. spicilegus* reared in SD photoperiod weighed less and investigated the novel object less than those reared in LD photoperiod. **(A)** Male *M. spicilegus* were reared in two different photoperiods. **(B)** Weight data from SD 14 h:10 h and LD 12 h:12 h reared male mice. SD males weighed significantly less than LD males (*p* = 0.024) **(C)** There was no significant difference in distance traveled in the open field between animals reared in SD and LD (*p* = 0.78). **(D)** There was no significant difference in time spent in the center of the open field between SD and LD (*p* = 0.28). **(E)** SD males contacted the novel object significantly less than LD males (*p* = 0.037). **(F)** SD males spent less time on top of the novel object than LD males (*p* = 0.037). These data demonstrate that photoperiod can significantly affect adolescent development in *M. spicilegus* reared in the laboratory. *p < 0.05 Mann-Whitney *U*-test.

In our final experiment we sought to use photoperiod to delay or blunt the increase in exploratory behavior in *Mus spicilegus*. We reasoned that if *M. spicilegus* born in SD autumnal photoperiod delay their dispersal in the wild, an autumnal photoperiod in the lab might delay an increase in exploratory behavior seen in the first months of life in LD conditions. To this end, we reared *M. spicilegus* in a SD 10 h:14 h light:dark cycle in the laboratory (Figure 3A).

Our first assay of mice reared in the SD protocol was body weight (the sample size for this group was larger than the sample size we were able to test in behavior). We found that SD reared mice showed significantly lower body weight than LD reared mice (SD 12.0 ± 0.25 g, *n* = 25; LD 13.3 ± 0.39 g, *n* = 35 (*U* = 288, *p* = 0.024) (Figure 3B).

We were able to test *n* = 13 male *M. spicilegus* reared on SD light and compare them to *n* = 29 male *M. spicilegus* reared on LD light (data shown above in age and sex comparisons). Contrary to our prediction, we found no significant difference between groups in distance traveled in the open field (*U* = 178, *p* = 0.78) and no significant difference in time spent in the center of the open field between males reared in LD and SD photoperiod (*U* = 148, *p* = 0.28) (Figures 3C,D). In the novel object test, P60-P79 male *M. spicilegus* reared in the SD photoperiod contacted the novel object for significantly less time than those reared in the LD photoperiod (*U* = 112, *p* = 0.037) (Figure 3E). Adolescent male *M. spicilegus* reared in SD photoperiod also spent significantly less time on top of the novel object than those reared in the longer photoperiod (*U* = 113, *p* = 0.037) (Figure 3F). These data establish novel object investigation was blunted by exposure to the SD photoperiod in male *M. spicilegus*.

## Discussion

Here we examined the maturation of exploratory behavior in lab based male and female *M. spicilegus* and tested if a photoperiod manipulation could blunt exploration behaviors. We found that distance traveled in the open field increased significantly between P21-P33 and P60-P79 in both male and female *M. spicilegus* reared in LD 12 h:12 h light:dark cycle in our lab. Robust novel object investigation was also observed in developing male and female *M. spicilegus* but did not show significant increases over the developmental time period we sampled. Our LD data showing increased in distance traveled are possibly consistent with motivation to disperse. In “the field,” *M. spicilegus* dispersal has been determined to occur in the first 2–3 months of life on long days (Lafaille et al., 2015) and in tests of exploratory behavior in the lab with *M. spicilegus* reared on a LD laboratory environment (Lafaille and Féron, 2014). However, alternate explanations for the observed developmental increases should also be considered (see more on this below).

When we compared male LD reared *M. spicilegus* behavior to C57 Bl/6 mice reared on the a 12 h:12 h light:dark cycle, we found *M. spicilegus* traveled longer distances than C57BL/6 lab mice; yet avoided the center of the empty open field more than their C57BL/6 counterparts. LD *M*. *spicilegus* were also bolder in their interactions with a novel object. Here species differences are also entwined with domestication history. We speculate less time spent in center in *M. spicilegus* reflect a strategic, predation-avoidant behavior that may have been lost over time in captivity in C57 Bl/6 mice.

Next, we used a photoperiod manipulation to test if rearing mice on SD light 10 h:14 h light: Dark in the lab was sufficient to blunt weight gain or exploratory behavior in male adolescent *M. spicilegus*. We found SD reared males achieved significantly lower weights at P60-70 and showed lower metrics of novel object investigation than LD reared *M. spicilegus*. These data suggest a photoperiod manipulation is sufficient to impact some aspects of physiology and behavior in *M. spicilegus* in the laboratory. This is an important first milestone and establishes that we can use photoperiod to regulate development in mice in the laboratory. This is significant because it is thought not to be possible in most inbred mouse lines due to their lack of melatonin (Kennaway, 2019).

While these results did uncover some developmental and photoperiod sensitive increases in exploratory behaviors, they did not fully meet the two pre-conditions we laid out for our goal. We found one set of behavioral metrics, open field distance traveled, that increased with development, but it was another set of metrics, novel object contact and mounting, that were blunted by SD photoperiod. We therefore cannot logically consider open field exploration or novel object investigation metrics as a stand-alone proxy for adolescent motivation to disperse. A battery of behavioral assays, or more ethological assays may serve as better indicators or markers.

As we develop a broader battery, we must also consider the emergence of behavioral changes related to mound building in *M. spicilgeus.* Given that wild living *M. spicilegus* born in the fall or winter months engage in mound building before overwintering and delaying dispersal until the following spring (Simeonovska-Nikolova and Mehmed, 2009; Szenczi et al., 2011; Hurtado et al., 2013), it is possible that adolescent SD *M. spicilegus* develop some form of exploratory behavior at the same age as LD *M. spicilegus*, to support mound building, or travel to mounds, but not dispersal over longer distances or toward reproductive ends. Again, further research using more ethological assays (see Hurtado et al., 2013) and photoperiod manipulations may help inform this question of the meaning of the expression of behavior in the lab.

### Lack of sex differences on long day photoperiod

The lack of sex differences in our LD experiments is also of significant interest as a finding. Previous studies have differed in their observation of sex differences in this species but this may be explained by age and light cycle (Garza et al., 1997; Simeonovska-Nikolova, 2000; Poteaux et al., 2008; Groo et al., 2013; Lafaille and Féron, 2014). In Groo et al. (2013) evaluated dispersal timing in LD 12 h:12 h light:dark laboratory conditions by modeling dispersal with an apparatus that required mice to cross a pool of water. Animals were tested up to the age of P120 and no significant differences between sexes were observed. Lafaille and Féron (2014) reared *M. spicilegus* in LD 14 h:10 h light:dark conditions and tested them in an open field test, elevated plus maze, and novel object investigation test. They found young mice (2 months) and old mice (24 months) to be similarly hyper-exploratory in both sexes when reared in constant LD summer-like photoperiod (Lafaille and Féron, 2014). However, by 6 months, females showed reduced exploration while male behavior remained unchanged. Simeonovska-Nikolova (2000) studied *M. spicilegus* caught directly from wild populations between spring and fall and found that males more actively explored their environment than females. The exact ages were unknown but all individuals were sexually mature adults. Since all animals were sexually mature, it is reasonable to assume they were over 2–3 months old and likely even older. These results may then corroborate exploratory measurements from Lafaille and Féron (2014) in suggesting exploration begins to decline with age earlier in females than in males for the cohort born in summer. In combination, these studies suggest that in *M. spicilegus*, the initial emergence of exploratory behavior gains for summer-born individuals is symmetric between sexes (in first 3–4 months), but may diverge at older ages.

Different mating systems are typically associated with different, sex specific dispersal trends (Dieckmann et al., 1999). *M. spicilegus* are primarily thought to be socially monogamous, as they exhibit characteristics such as pair bonding, considerable olfactory bulb neurogenesis in response to their mate, biparental care of young, and heightened aggression toward unfamiliar conspecifics (Patris and Baudoin, 2000; Baudoin et al., 2005). Others have argued for a polygynous mating system in *M. spicilegus*, though evidence for this has only been observed in the early spring, directly following dispersal (Gouat et al., 2003a; Bardet et al., 2007; Poteaux et al., 2008). It is possible that the sex differences in dispersal behavior in the LD born *M. spicilegus* cohort differ from the SD overwintering *M. spicilegus*. There is good evidence of male biased dispersal from mounds following overwintering in the wild (Greenwood, 1980; Poteaux et al., 2008; Simeonovska-Nikolova, 2012; Csanády et al., 2020). Genetic studies of wild populations have shown that females remain near the mounds after overwintering while males disperse greater distances (Poteaux et al., 2008; Simeonovska-Nikolova, 2012). Comparable comprehensive genetic studies of the summer cohort dispersal dynamics have not been conducted to our knowledge. Based on these data, we predict that sex differences may emerge in a laboratory based study of *M. spicilegus* raised on SD light cycles at older ages.

We also consider that social factors may also contribute to seasonal sex differences in dispersal. The sexual maturity of females has been found to be impacted by social environment (Féron and Gheusi, 2003; Gouat et al., 2003b). In the summer, females are particularly aggressive toward other females, and pregnant females demonstrate heightened levels of aggression compared to non-pregnant females (Simeonovska-Nikolova, 2012; Ambaryan et al., 2019). In the summer, there is no evidence of group living or cooperation other than between mated pairs (Milishnikov et al., 1998). In the wild, these changing environmental and social conditions may differentially impact males and females and social partners may need to be included to capture the role of these influences on studies dispersal in the laboratory.

## Limitations and future directions

Our study used highly simplified metrics of exploration to assay behavior. More ethological tasks have been invented to study *M. spicilegus* behavior (Groo et al., 2013; Hurtado et al., 2013) and could be applied with photoperiod manipulation to test how photoperiod regulates dispersal related behavior in laboratory conditions. Future experiments may also consider: (1) More accurate mimicry of season through longer LD day length, changing day length over time and including a temperature manipulation as well as a photoperiod manipulation; (2) studying behavior over time periods longer than 3 months, (3) studying females more closely; and (4) allowing social factors to influence behavior. Furthermore, measures of gonadal hormonal status and corticosterone may also help inform our understanding of adolescent development and photoperiod in *M spicilegus.*

## Conclusion

We examined the adolescent development of exploratory behavior *M. spicilegus*, a mouse model which may offer an exciting opportunity to use photoperiod to control the emergence of dispersal behavior. In this wild-derived mouse reared on LD photoperiods, we observed high levels of novel object investigation at juvenile and adolescent ages and robust increases in exploratory behavior in the open field over the first 3 months of life. When we manipulated photoperiod to mimic SD “autumnal” conditions that delay dispersal in the wild, we found we significantly blunted weight gain and novel object investigation metrics, but not open field metrics. These data establish that photoperiod can impact the adolescent development of male *M. spicilegus* weight and exploratory behavior in captivity. The relevance of these changes to dispersal and mound building behavior will need to be informed by further work using more ethological assays.

## Data availability statement

The raw data supporting the conclusions of this article are available in Supplementary materials.

## Ethics statement

This animal study was reviewed and approved by the Animal Care and Use Committee, University of California, Berkeley.

## Author contributions

NC conceptualized experiments, ran the experiments, analyzed the data, and wrote the manuscript. GP and WL conceptualized experiments, ran experiments, supervised experiments, and contributed to the analyses and manuscript. OK, WC, and NM ran experiments and contributed to the analyses and manuscript. LW conceptualized and supervised experiments, data analyses, and wrote the manuscript. All authors contributed to the article and approved the submitted version.
